# A diverse parasite pool can improve effectiveness of biological control constrained by genotype‐by‐genotype interactions

**DOI:** 10.1111/eva.13501

**Published:** 2022-11-05

**Authors:** Fabiane M. Mundim, Amanda K. Gibson

**Affiliations:** ^1^ Department of Biology University of Virginia Charlottesville Virginia USA

**Keywords:** coevolution, genetic diversity, genotype‐by‐genotype interactions, host–parasite interactions, *Meloidogyne*, *Pasteuria*

## Abstract

The outcomes of biological control programs can be highly variable, with natural enemies often failing to establish or spread in pest populations. This variability has posed a major obstacle in use of the bacterial parasite *Pasteuria penetrans* for biological control of *Meloidogyne* species, economically devastating plant‐parasitic nematodes for which there are limited management options. A leading hypothesis for this variability in control is that infection is successful only for specific combinations of bacterial and nematode genotypes. Under this hypothesis, failure of biological control results from the use of *P. penetrans* genotypes that cannot infect local *Meloidogyne* genotypes. We tested this hypothesis using isofemale lines of *M. arenaria* derived from a single field population and multiple sources of *P. penetrans* from the same and nearby fields. In strong support of the hypothesis, susceptibility to infection depended on the specific combination of host line and parasite source, with lines of *M. arenaria* varying substantially in which *P. penetrans* source could infect them. In light of this result, we tested whether using a diverse pool of *P. penetrans* could increase infection and thereby control. We found that increasing the diversity of the *P. penetrans* inoculum from one to eight sources more than doubled the fraction of *M. arenaria* individuals susceptible to infection and reduced variation in susceptibility across host lines. Together, our results highlight genotype‐by‐genotype specificity as an important cause of variation in biological control and call for the maintenance of genetic diversity in natural enemy populations.

## 1 INTRODUCTION

Host populations show substantial genetic variation in their susceptibility to parasites, and parasite populations likewise show considerable genetic variation in their infectivity (Laine et al., 2011; Tack et al., 2012; Thompson, 1988). Critically, the outcome of infection often does not depend on host and parasite traits in isolation, but on the specific interaction of host and parasite genotype (i.e., a genotype‐by‐genotype or GxG interaction) (e.g., Carius et al., 2001; Lively, 1989; Parker, 1985; Schmid‐Hempel, 2001). Genotype‐by‐genotype interactions may dictate the success of biological control, in which natural enemies, like parasites, are used to suppress populations of pest species (Holt & Hochberg, 1997; Mackauer, 1976; Roderick & Navajas, 2003).

The outcomes of biological control programs are highly variable, which poses a great challenge to their widespread implementation. Variable outcomes have been attributed to stochastic loss of small enemy populations (Hopper & Roush, 1993) and variation in the suitability of the local environment or pest species for the released enemies (Henry et al., 2010; McDonald, 1976; Messenger & van den Bosch, 1971; van Klinken et al., 2003; Zepeda‐Paulo et al., 2013). However, variation in the interaction of natural enemy genotypes with local pest genotypes could also contribute strongly to the observed variability in biological control outcomes (Hopper et al., 1993; Hufbauer & Roderick, 2005). For example, genotypes of the parasitoid wasp *Lysiphlebus fabarum* vary in which genotypes of the aphid *Aphis fabae* they can infect. This genotype‐by‐genotype specificity is mediated by the ability of a wasp genotype to overcome protection conferred by an aphid clone's strain of the defensive symbiont *Hamiltonella defensa* (Cayetano & Vorburger, 2013; Rouchet & Vorburger, 2014). Biological control of experimental aphid populations was improved by first selecting wasp populations to overcome symbiont strains common in the aphid populations (Rossbacher & Vorburger, 2020). Studies have also found evidence of genotype‐by‐genotype interactions by comparing the performance of biological control agents on hosts from different locations. For example, phytophagous mites (*Floracarus perrepae*) were more effective at attacking haplotypes of the invasive fern *Lygodium microphyllum* from their same geographic region than from distant regions (Goolsby et al., 2006). Likewise, some strains of the parasitoid wasp *Cotesia typhae* parasitize local strains of their host, the corn borer *Sesamiae nonagroides*, at higher rates than foreign host strains (Benoist et al., 2020). This body of work demonstrates the potential significance of genotype‐by‐genotype interactions, but the scarcity of such studies leaves us uncertain of their general relevance in biological control.

If genotype‐by‐genotype interactions determine infection outcomes, then consistent biological control can be achieved only if management programs are designed to accommodate this specificity. One solution is to tailor biological control agents to specific pest populations, either by identifying parasite populations with high performance (e.g., Benoist et al., 2020; Goolsby et al., 2006) or generating them in captivity via artificial selection against the target pest population (e.g., Rossbacher & Vorburger, 2020). Another solution is to initiate biological control with a genetically diverse population of parasites (Szűcs et al., 2019). If genotype‐by‐genotype interactions determine infection success, then increasing the genetic diversity of the parasite population should increase the success of pest control by increasing the probability that a pest host encounters a parasite genotype that can infect it (Ganz & Ebert, 2010; Gibson, 2021; van Baalen & Beekman, 2006). For example, administering a cocktail of multiple bacteriophages can increase control of bacteria infecting humans, livestock, and crop plants (Lin et al., 2017; Svircev et al., 2018; Wittebole et al., 2014). Beyond bacteriophages, increasing parasite diversity has rarely been tested as a strategy for improving biological control in the face of genotype‐by‐genotype interactions (see Channer & Gowen, 1992).

Genotype‐by‐genotype specificity may be a critical factor explaining variation in the efficacy of the bacterial parasite *Pasteuria penetrans* in biological control of root‐knot nematodes (*Meloidogyne*). Root‐knot nematodes are obligate, sedentary endoparasites of a wide range of crops, including soybean, cassava, tomato, and many other vegetables (Jones et al., 2013; Nicol et al., 2011; Onkendi et al., 2014). Infective juveniles (J2s) penetrate plant roots and migrate to the vascular tissue, where they establish feeding sites and stimulate gall formation. When females reach reproductive maturity, they deposit egg masses on the root surface, and the next generation of J2s hatches into the soil (Shurtleff & Averre, 2005). Root‐knot nematodes cause billions of dollars in damage every year by siphoning nutrients from plants and predisposing them to other infections (Hua et al., 2019; Morris et al., 2016). Rising temperatures will likely increase their geographic range (Ghini et al., 2008), and effective nematicides are increasingly scarce due to their environmental toxicity (Zasada et al., 2010). Thus, there is an urgent, global demand for new control strategies.

*Pasteuria penetrans* has been considered as a potential biological control agent since the 1970s (Mankau, 1975; Mankau & Imbriani, 1975), because it is a natural, obligate parasite that limits *Meloidogyne* reproduction (Davies, 2009). Infection starts with the external adhesion (attachment) of *P. penetrans* endospores to the cuticle of J2s as they migrate through soil (Sayre & Wergin, 1977). Attached endospores hinder mobility, which can prevent nematodes from finding and entering a plant root (Vagelas et al., 2012). If a J2 succeeds in entering a plant root, four to 10 days later the attached endospores germinate, sending a germinal tube through the cuticle of the nematode into its pseudocoelom. *Pasteuria penetrans* then grows vegetatively, filling the body cavity with endospores and reducing nematode reproduction. Endospores are released into the soil upon disintegration of the nematode cuticle (Chen & Dickson, 1998). *Pasteuria penetrans* can strongly reduce *Meloidogyne* density (e.g., > 80% in Bhuiyan et al., 2018) and reduce crop damage (e.g., Timper et al., 2016). In field trials, however, *P. penetrans* and other *Pasteuria* species vary substantially in their ability to control nematode populations (Bissonnette et al., 2018; Kariuki & Dickson, 2007).

The leading hypothesis for this variability is that infection depends on the specific interaction of host and parasite genotype (Channer & Gowen, 1992; Timper, 2009). A single isolate of *P. penetrans* can infect some *Meloidogyne* lines but not others (Stirling, 1985; Trudgill et al., 2000), with isolates differing in which lines they can infect (Liu et al., 2018; Timper, 2009). This hypothesis has support from the related bacterial parasite *P. ramosa* and its crustacean host *Daphnia magna*: the outcome of infection in this system rests upon a strong genotype‐by‐genotype interaction that manifests at the attachment stage (Carius et al., 2001; Duneau et al., 2011; Luijckx et al., 2011, 2013). The working model proposes that host genotypes vary in the receptors on their cuticle to which *Pasteuria* endospores must attach to initiate infection. If *Pasteuria* genotypes in turn vary in the binding proteins on their endospore surface, then infection outcomes may rest on the compatibility of host and parasite proteins at this attachment stage (Davies, 2009; Davies & Opperman, 2006). Further evaluation of this model for *P. penetrans* and *Meloidogyne* requires that genetic specificity in this system be fully characterized. Currently, we have little sense of the extent and nature of genetic variation for susceptibility to *P. penetrans* within *Meloidogyne* species. If susceptibility indeed depends on the specific interaction of host and parasite genotype, then successful control may require either prior selection of *P. penetrans* for performance on locally common *Meloidogyne* genotypes or the use of genetically diverse cocktails of *P. penetrans*, a**s** proposed by Channer and Gowen (1992).

The objectives of this study were to (1) evaluate the significance of genotype‐by‐genotype interactions in infection of *Meloidogyne* by *P. penetrans*, and (2) test the use of genetically diverse parasite populations as a strategy for improving the success of biological control. First, we determined if attachment depends on the specific interaction of host and parasite genotype by establishing 13 isofemale lines of the peanut root‐knot nematode *M. arenaria* from a single population and exposing them to four sources of *P. penetrans*. Second, we asked if genetically diverse parasite populations have higher attachment by exposing four isofemale lines of *M. arenaria* to populations of *P. penetrans* with low and high diversity. Our results provide specific guidance for the management of *M. arenaria* with *P. penetrans* and lend weight to genotype‐by‐genotype interactions as an important consideration for ensuring consistently effective biological control programs.

## 2 MATERIALS AND METHODS

### 2.1 Establishment of host and parasite lineages

We established 13 isofemale lines of *Meloidogyne arenaria* from egg masses on peanut roots (*Arachis hypogaea*) collected from the Tubbs field at the University of Georgia Gibbs Farm in Tifton, GA, USA. The peanut roots were washed, cut into 3–4 cm lengths, and placed in a solution of 20% red food coloring (McCormick®) for 15 min. The food coloring stains egg masses bright red, allowing us to use a stereoscope to easily locate them on the root surface (Thies et al., 2002; Appendix S2.1). *Meloidogyne arenaria* reproduces by mitotic parthenogenesis (Marais & Kruger, 1991; Triantaphyllou, 1962, 1981), so offspring from one egg mass is genetically identical descendants of one mother. Therefore, to establish an isofemale line, we inoculated an eggplant (*Solanum melongena*) with a single egg mass. Isofemale lines were left to proliferate on eggplants for three months in the greenhouse under 16 h of daylight at an average temperature of 29°C during the day and 21°C at night (Appendix S2.2). They were transferred to new eggplants approximately every three months. All lines were reared in the same environment to ensure that variation in attachment phenotypes reflected genetically‐based differences.

To capture the natural genetic variation of *P. penetrans* with which our host population was likely to interact, we collected *P. penetrans* endospores from the Tubbs field and seven additional fields in Tifton that were previously identified as having large *P. penetrans* populations. These seven fields included a second field at the Gibbs Farm, two fields at the Black shank Farm, one field at the Bowen Holbrook Farm, and three fields at the Lang Farm. The fields are within 10 miles of one another (Appendix S2.3). We collected soil by taking eight cores (15–20 cm depth) from a 1x1.8‐m square area in a clockwise manner from the field entry point. We let the soil dry at room temperature in the lab for three days and then stored the soils at 4°C until the start of the experiment. Each parasite source was expected to contain a mixture of *P. penetrans* genotypes, and the abundance of endospores (i.e., dose) may have varied between sources.

### 2.2 Experiment 1: Evaluating the significance of GxG interactions

The objective of this first experiment was to determine if attachment varies with the interaction of host line and parasite source. To do so, we measured attachment of four of our *P. penetrans* sources to our 13 *M. arenaria* isofemale lines.

We obtained J2s for each isofemale line by extracting eggs from the roots of eggplants using an adaptation of the Oostenbrink method (Oostenbrink, 1960; Appendix S2.4). We allowed the eggs to hatch in water for up to 72 h, collected the J2s, and maintained these J2 suspensions at 4°C for no more than 5 days. We obtained endospores from each parasite source by saturating dried soil with water and then sieving out particulate debris to retain a suspension of endospores (Appendix S2.5). For each isofemale line, we brought the J2s to room temperature and added 10 ml of the J2 suspension to each replicate flask for that line. We then added the appropriate endospore suspension to bring the final volume per flask to 200 ml. Flasks were agitated on a rotatory shaker at 180 rpm for 48 h at room temperature to facilitate contact between J2s and endospores. We then extracted J2s from the flasks by centrifugal flotation (Appendix S2.6; Timper et al., 2001). Each combination of host line and parasite source was replicated six times (i.e., six flasks).

We measured attachment rate and load of J2s in each flask at a magnification of 10 – 40x on an inverted microscope. Attachment rate is the percentage of nematodes with one or more endospores attached. This measurement captures variation between host–parasite combinations in the percentage of hosts susceptible to infection. To measure attachment rate, we counted the number of nematodes with and without endospores for an average of 17.45 ± 0.70 (standard error) nematodes per flask. For one host line (H02), we had fewer nematodes per flask (< 10). A second measurement, attachment load, is the number of endospores attached per nematode. Attachment load is estimated only for hosts with one or more endospores attached. Thus, attachment load can capture variation between host–parasite combinations in the number of parasites that hosts acquire, given that a host line is susceptible to infection by a parasite source (i.e., attachment rate >0). To measure attachment load, we counted the number of endospores attached to the cuticle of up to 15 nematodes with endospores per flask. We were unable to isolate nematodes from a subset of flasks for three host lines; these flasks were excluded from all analyses (H02: 7/24; H05: 12/24; H07: 7/24 flasks excluded).

### 2.3 Experiment 2: Evaluating the effect of parasite diversity on infection probability

The objective of this experiment was to determine if increasing parasite diversity increases attachment rate. We used all eight *P. penetrans* sources (Appendix S2.3) and four host lines (H04, H08, H10, H13) that varied from low to high mean attachment rates in Experiment 1. We used the eight individual sources of *P. penetrans* as our low‐diversity treatment (P01 – P08). We created a high‐diversity parasite source (MIX) by combining equal volumes of soil from each of the eight individual sources. This approach created parasite treatments that varied qualitatively in diversity; we did not quantify the diversity of the high vs. low‐diversity treatments, because we did not perform genetic analyses of field‐sampled parasite sources. We tested each host line against the eight low‐diversity parasite sources and the high‐diversity parasite source, inoculating with equal volumes of soil across diversity treatments. The high‐diversity flasks therefore had an equal abundance (dose) of endospores as the single‐source flasks on average. Each host–parasite combination was replicated in four flasks. We measured attachment rate and load as above. For attachment rate, we collected data from 21.71 ± 0.73 nematodes per flask.

### 2.4 Statistical analyses

We performed all the analyses in R v4.1.1 (R Core Team, 2019). We used AIC and BIC criteria to compare models, and the *emmeans* function for all Tukey's post hoc multiple pairwise comparisons (Lenth et al., 2019). For Experiment 1, we included host line, parasite source, and their interaction as fixed effects. The main effect of parasite source controls for the effect of intrinsic variation in dose on attachment. Thus, the interaction effect reflects variation in the susceptibility of host lines to different parasite sources that is not attributable to differences in dose. To evaluate variation in attachment rate, we fit generalized linear models with a binomial distribution to the number of nematodes with and without endospores attached per flask. To evaluate variation in attachment load, we excluded hosts with no endospores attached and used the *glmer* function from the lmer4 package (Bates et al., 2015) to fit generalized linear mixed‐effects models with a Poisson distribution to the number of endospores per host. We included a unique identifier (ID) for each nematode as a random effect to correct for overdispersion. We initially included flask as a random effect, but the model was equivalent to that with ID alone, so we proceeded with the simpler model (*p* = 0.99).

For Experiment 2, we included diversity treatment (high or low) as a fixed effect and host line and parasite source as random effects. To evaluate variation in attachment rate, we fit a generalized linear mixed‐effects model with a binomial distribution to the number of nematodes with and without endospores attached per flask. We included flask as a random effect to correct for overdispersion. To evaluate variation in attachment load, we fit a generalized linear mixed‐effects model with a Poisson distribution to the number of endospores per host. We included flask as a random effect to account for repeated measures. In addition, to validate the results of Experiment 1, we excluded data from the high‐diversity treatment and again tested for an interaction effect of *P. penetrans* single source with host line on variation in attachment rate and load. We fit models as described for Experiment 1, including host line, parasite source (P01 – P08), and their interaction as fixed effects.

## 3 RESULTS

### 3.1 Does infection probability vary with the interaction of host line and parasite source?

**FIGURE 1:**
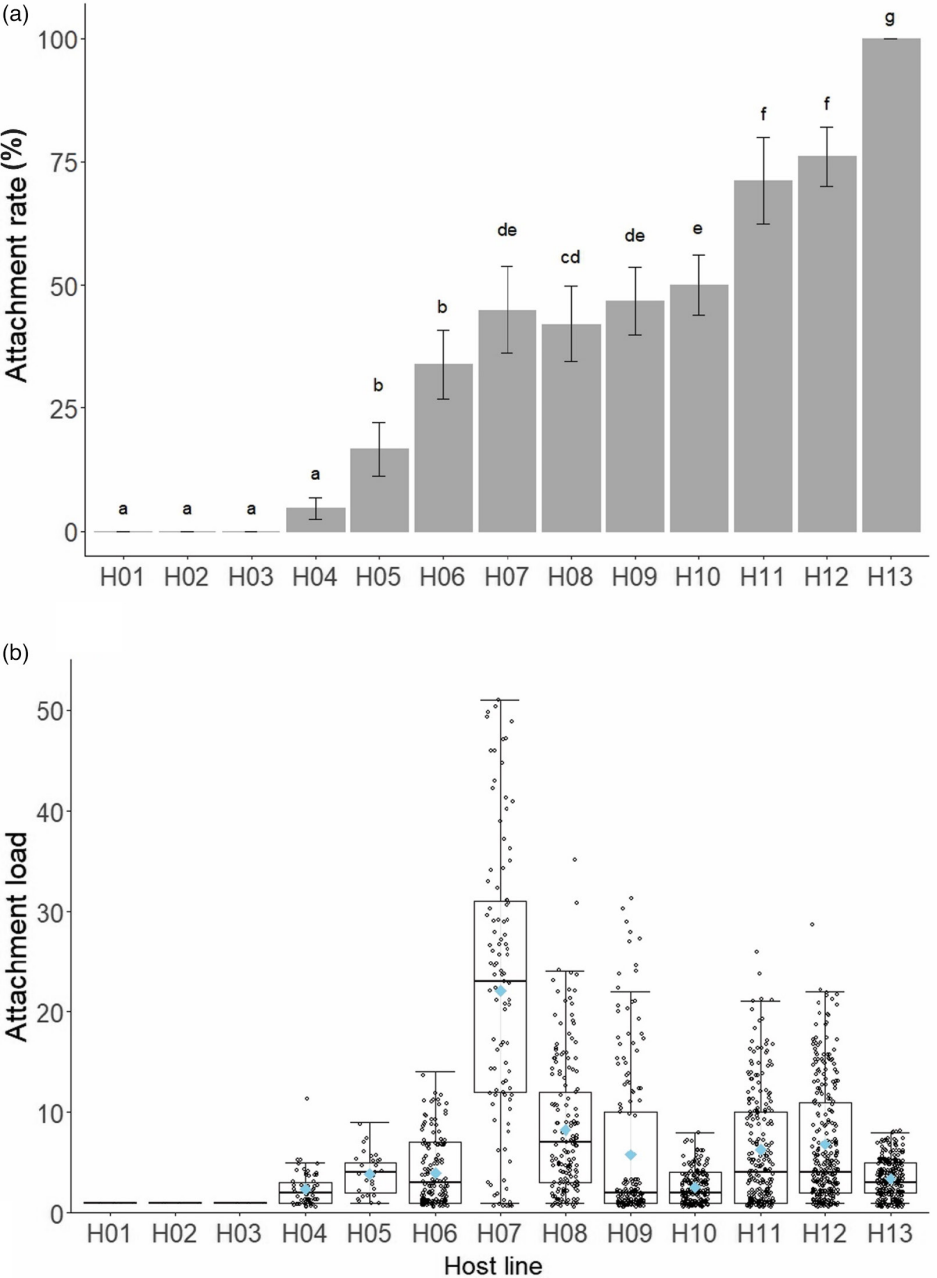
Attachment rate and load varies with host genotype. (a) Attachment rate is the percentage of hosts with endospores attached to their cuticle. The bars represent means across replicates, and error bars indicate standard error. Different letters indicate significant differences among host lines (*p* < 0.05) based on post hoc Tukey's tests. (b) Attachment load is the number of endospores attached to the cuticle of hosts that had one or more endospores attached. The boxes represent the interquartile range, the horizontal black lines indicate the medians, whiskers extend to 25% and 75% quartiles. Black points represent load for individual hosts, and the blue diamond indicates the mean. For both (a) and (b), each host line was tested against four parasite sources with six replicates per source (i.e., 24 replicate flasks per host line).

In our first experiment, we tested whether the critical first step in the infection process, attachment, varies with the interaction of host line and parasite source. We found that both host line, parasite source, and their interaction explained variation in attachment of *P. penetrans* to *M. arenaria* (Table 1). Host lines varied in attachment rate independently of parasite source (χ^2^ = 2264.5; *df* = 12; *p* < 0.001; Table S1a; Figure 1a). Three host lines had no endospores attached across all four parasite sources (H01 – H03), while for one host line, 100% of individuals had endospores attached (H13). Host lines also varied in attachment load independently of parasite source (χ^2^ = 1425.9; *df* = 9; *p* < 0.001; Table S1b; Figure 1b). For hosts with endospores attached, the mean number attached was 6.23 ± 0.18 (standard error). H07 had the highest mean load, with 22.25 ± 1.38 endospores per host, while H04 had the lowest mean load with 2.42 ± 0.24.

**TABLE 1:** Variation in attachment rate and load according to host genotype, parasite source, and their interaction.

Model	Df	AIC	ΔAIC	Weight	BIC
a. Attachment rate					
Host line * Parasite source	**49**	**654.2**	**0**	**1.00**	**833.3**
Host line + Parasite source	16	2003.3	1349.1	0.00	2061.8
Host line	13	2222.1	1567.9	0.00	2269.7
Parasite source	4	4243.8	3589.6	0.00	4258.4
b. Attachment load					
Host line * Parasite source	**32**	**7515.1**	**0**	**1.00**	**7688.4**
Host line + Parasite source	14	8895.5	1380.4	0.00	8971.3
Host line	11	8945.0	1429.9	0.00	9004.6
Parasite source	5	9348.6	1833.5	0.00	9375.7

*Note*: Model comparisons for (a) attachment rate, the percentage of hosts with endospores attached, and (b) attachment load, the number of endospores attached per host with one or more endospores attached. The best model based on AIC and BIC is denoted in bold, and Weight refers to Akaike weight, a measure of the relative likelihood of the model.

**FIGURE 2:**
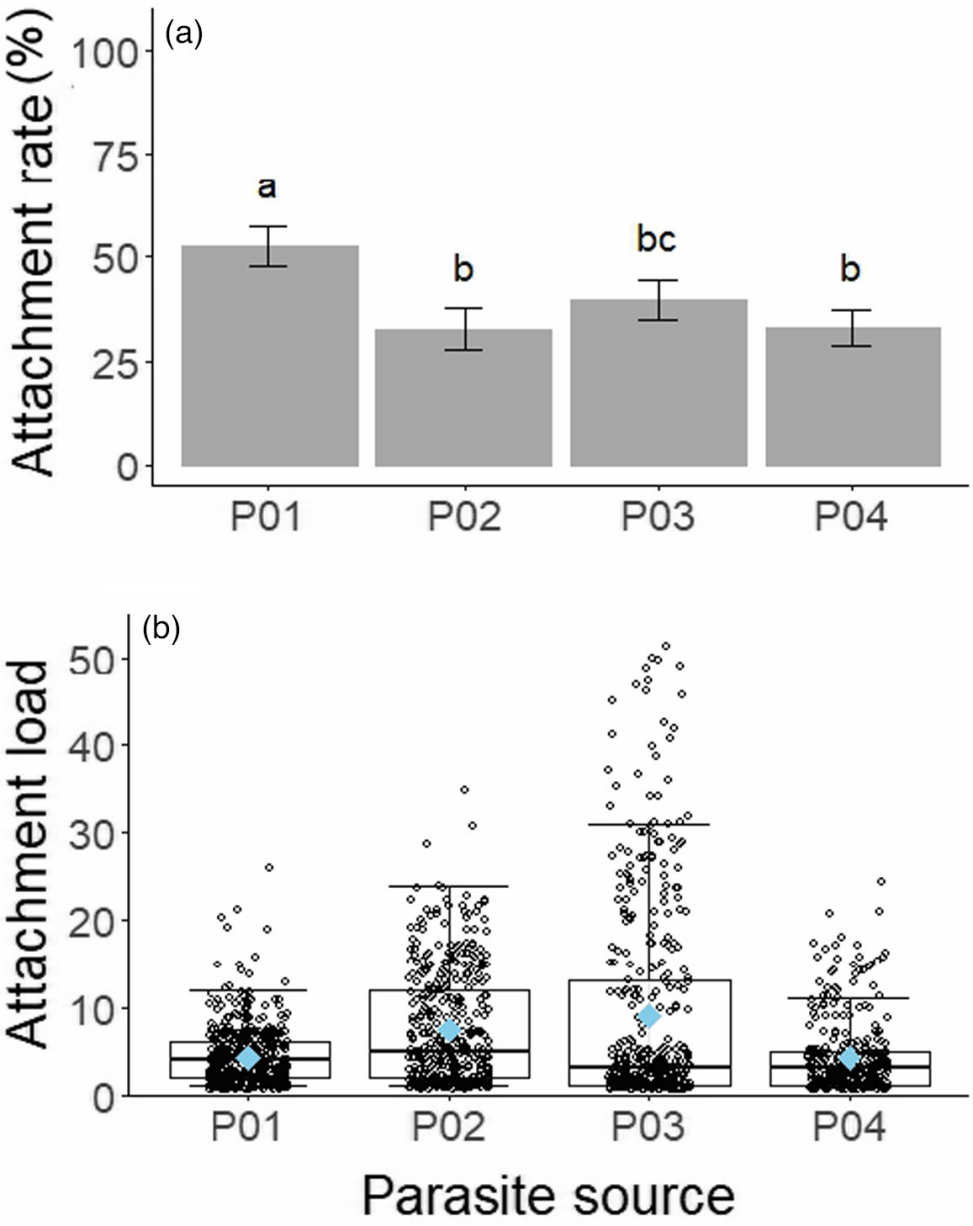
Attachment rate and load varies with parasite source. (a) Attachment rate and (b) load are estimated and represented as in Figure 1, with means taken across replicate flasks (six replicate flasks x 13 isofemale lines = 78 flasks per parasite source).

Parasite sources varied moderately in attachment rate and load independently of host line (rate: χ^2^ = 224.8; *df* = 3; *p* < 0.001; load: χ^2^ = 223.8; *df* = 3; *p* < 0.001; Table S1, Figure 2). Across sources, mean attachment rate ranged from 32.57 ± 4.99% (P02) to 52.66 ± 4.94% (P01), and mean load varied from 4.26 ± 0.23 (P04) to 8.89 ± 0.57 (P03) endospores per host. This variation among parasite sources could reflect intrinsic variation in attachment ability or in endospore dose.

**FIGURE 3:**
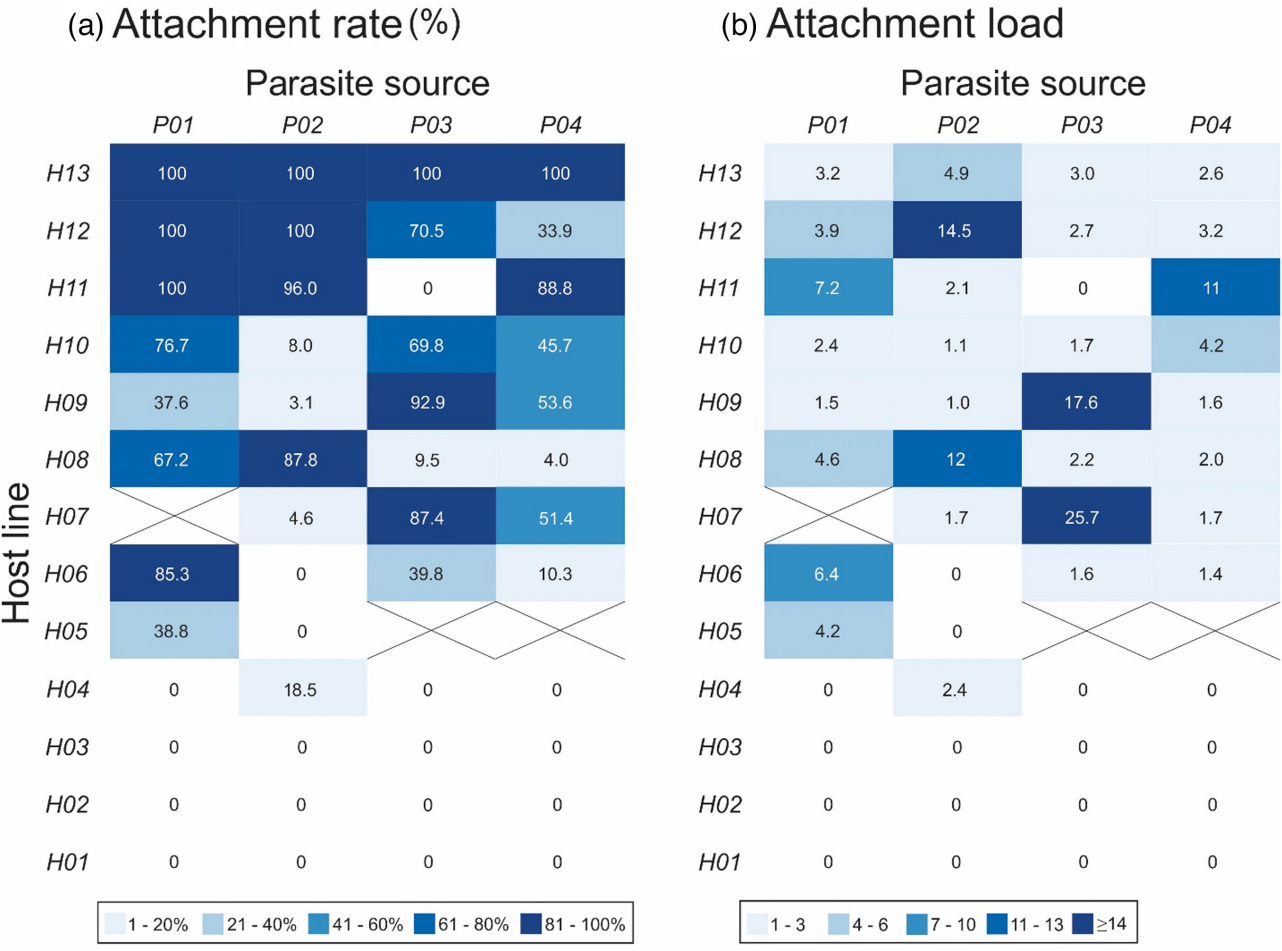
Attachment rate and load varies with the combination of host and parasite. (a) Attachment rate and (b) load for each combination of host line and parasite source. The cells are shaded such that darker colors indicate relatively high attachment rate or load. The number in each cell gives the combination's (a) mean percentage of hosts with endospores attached to the cuticle and (b) the mean number of endospores attached per host with one or more endospores attached. Crossed cells indicate combinations for which we had no data. For a more detailed presentation of the data, see Figure S1 for (a) and Figure S2 for (b).

The interaction of host line and parasite source contributed strongly to variation in attachment rate and load (rate: χ^2^ = 1415.1; *df* = 33; *p* < 0.001; load: χ^2^ = 1803.9; *df* = 18; *p* < 0.001; Table S1; Figure 3). A given host line's attachment rate tended to vary substantially with parasite source, with some host lines susceptible to attachment by only a subset of parasite sources (Figure 3a, Figure S1). No parasite source was able to attach to all host lines, and parasite sources differed in which host lines they could attach to. Attachment load similarly varied with the interaction of host line and parasite source (Figure 3b, Figure S2), with most host lines showing high attachment load with one parasite source but not another, and vice versa.

### 3.2 Does increasing parasite diversity increase infection probability?

**FIGURE 4:**
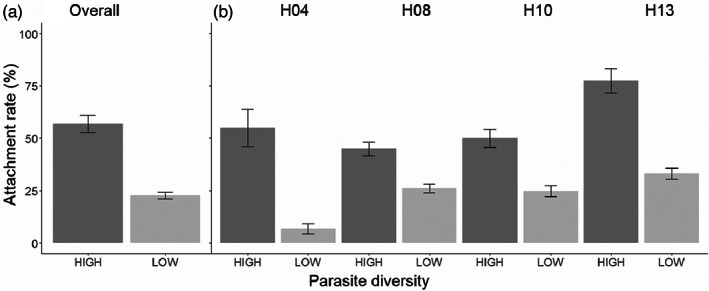
Increasing parasite diversity increases attachment rate. Each of four host lines was exposed to eight low‐diversity parasite populations (single parasite sources) and one high‐diversity parasite population (eight parasite sources combined). Each combination was tested in four replicates with up to 30 hosts examined per replicate. Panel (a) shows the mean attachment rate according to parasite diversity level, and panel (b) breaks this down according to individual host lines. Error bars show standard error of the mean. See Figure S3 for further detail.

Given this evidence for a substantial interaction effect underlying the probability of attachment of *P. penetrans* to *M. arenaria* (Figure 3a, Figure S1), we predicted that increasing the diversity of the parasite population would increase attachment rate by increasing the probability that a host line encounters a parasite genotype to which it is susceptible. Consistent with this prediction, we found that the high‐diversity parasite source had a significantly higher attachment rate than the low‐diversity parasite sources (coefficient = 1.6 ± 0.3, *z* = 4.7, *p* < 0.001, Table S2a, Figure 4). The high‐diversity source attached to 56.87 ± 4.17% of hosts, 2.5‐fold more than the average of the low‐diversity sources (22.66 ± 1.45%). The high‐diversity source attached to all four tested host lines, with attachment rate ranging from 45.00 ± 3.19% (H08) to 77.50 ± 5.68% (H13) of hosts. In contrast, the low‐diversity sources varied substantially in their ability to attach to a given host line: some low‐diversity sources attached at a rate statistically indistinguishable from the high‐diversity source, while other low‐diversity sources attached to few to no hosts (Table S3a; Figure S3).

The high‐diversity and low‐diversity sources did not differ in attachment load (coefficient = 0.2 ± 0.2, *z* = 0.9, *p* = 0.376, Table S2b). Attachment load was generally lower than in the first experiment: hosts had 2.41 ± 0.10 endospores attached with the high‐diversity source, vs. 2.27 ± 0.08 with the low‐diversity sources. Low‐diversity sources had lower load than the high‐diversity source in some pairings, but for each host line, the maximum load was found in combination with a low‐diversity source rather than with the high‐diversity source (Table S3b, Figure S4).

Consistent with the results of the first experiment, the interaction of host line and low‐diversity parasite source contributed strongly to variation in attachment rate and load (Table S4).

## 4 DISCUSSION

This study tests the hypothesis that genetic interactions between natural enemies and their target pests can create variation in the outcome of biological control. Consistent with this idea, we found that the ability of the biological control agent *P. penetrans* to attach to, and thereby infect, the plant pest *M. arenaria* depends on the interaction of host genotype and parasite source (Figure 3). Given this result, we tested the use of genetically diverse parasite populations as a tool for improving biological control outcomes. We found that increasing the genetic diversity of the *P. penetrans* population increased the potential to control *M. arenaria* populations by nearly doubling mean attachment rate relative to *P. penetrans* populations with lower diversity (Figure 4).

Our first experiment demonstrated that the rate and load of endospore attachment varied with the genotype of *M. arenaria* (Figure 1), the source of *P. penetrans* (Figure 2), and their interaction (Figure 3). Our second experiment further validated these results (Table S4). We address each of these factors in order. First, the main effect of host line tells us that *M. arenaria* genotypes vary in their susceptibility to attachment, irrespective of the genotypes of *P. penetrans* to which they were exposed (Figure 1). Because these 13 host lines were isolated from a single field, this result indicates that a population of *M. arenaria* can maintain many phenotypically distinct asexual lineages. This result expands the findings of Timper (2009), which reported variation in attachment among five *M. arenaria* lines from our study population. Together, these findings establish extensive within‐population variation for a phenotype relevant to biological control of this problematic plant parasite. Notably, three host lines were resistant to attachment by all parasite sources tested, indicating the potential for rapid evolution of resistance to *P. penetrans* in *M. arenaria* populations. Resistance, however, is a function of parasite genotype (Figure 3), and we suspect that testing additional sources of *P. penetrans* would increase attachment to these seemingly resistant host lines (Frank, 1994).

The main effect of parasite source tells us that our populations of *P. penetrans* differed in attachment rate and load, irrespective of the host genotype on which they were tested (Figure 2). In using populations of *P. penetrans* sampled directly from our field sites, we captured the local diversity of parasites encountered by our host population. This approach, however, prevented standardization of endospore abundance (i.e., dose) and rearing environment across parasite sources. Thus, our data cannot establish whether intrinsic variation in attachment among parasite sources is due to variation in dose, environmental effects, or genetic composition. Variation in dose is the most obvious difference between parasite sources. Parasite sources with the highest mean attachment rate did not, however, have the highest mean load (Figure 2a,b), which we would expect if variation among parasite sources was attributable solely to dose. We do not have a clear sense of how environmental conditions in the field differed for our *P. penetrans* populations. Importantly, we treated parasite sources identically from the point of collection, including during attachment assays, as conditions at this stage are known to influence attachment (Chen & Dickson, 1998). Thus, we expect that the differences among parasite sources derive in part from variation in their genetic composition, consistent with prior surveys of *P. penetrans* (Joseph et al., 2017; Stirling, 1985; Trudgill et al., 2000). We observed less variation among parasite sources than among host lines (Figure 1 vs. 2). This is likely due to the fact that the parasite sources contained multiple distinct genotypes of *P. penetrans*, reducing the potential for variation among parasite sources. Accordingly, we would expect increased variation following establishment of clonal lines of *P. penetrans* (Luijckx et al., 2011).

Critically, our first experiment established that the interaction of host genotype and parasite source is a major driver of variation in attachment rate and load. We found that the same host line could be highly susceptible to attachment by one parasite source and resistant to attachment by another (Figure 3a). Similarly, the same parasite source may attach at high rates to one host line but fail to attach to another (Figure 3a) and vary dramatically in mean load across host lines, in spite of an equivalent dose of endospores (Figure 3b). Moreover, two parasite sources could have similar attachment rates on a given host genotype, but very different attachment loads (Figure 3a,b; e.g., P01 and P02 on H11 or H12). These results build upon prior studies in which lines established from the major *Meloidogyne* species (typically *M. incognita*, *M. arenaria*, and *M. javanica)* varied in the *P. penetrans* isolates to which they were susceptible. These studies typically used *Meloidogyne* and *P. penetrans* genotypes collected from distinct populations, often from different countries or continents, so it has been unclear whether this variation in susceptibility reflected differentiation among geographically isolated populations or the maintenance of variation at a microevolutionary scale (Channer & Gowen, 1992; Davies et al., 2001; Stirling, 1985; Trudgill et al., 2000). We paired host lines from a single population of *M. arenaria* with geographically overlapping collections of *P. penetrans* genotypes, thereby establishing that variation in susceptibility across host–parasite combinations is maintained at a local scale.

Our results coincide with findings from *P. ramosa* and *D. magna*, a prominent system for the study of host–parasite coevolution. The *P. ramosa–D. magna* system shows substantial variation within and between populations in host susceptibility and parasite infectivity (Carius et al., 2001; Ebert et al., 1998). Variation in infection outcomes stems largely from a strong genotype‐by‐genotype interaction governing susceptibility at the attachment step (Duneau et al., 2011; Luijckx et al., 2011). Attachment is hypothesized to depend on the ability of collagen‐like adhesion proteins on the surface of *P. ramosa* endospores to bind to proteins on the surface of the *D. magna* cuticle. The genes encoding these collagen‐like proteins are immensely polymorphic in *P. ramosa* (McElroy et al., 2011; Mouton et al., 2009), and for at least one of these genes, the polymorphisms alter the protein structure at the point of contact with the host cuticle (Andras et al., 2020). Collagen‐like proteins also play a critical role in *P. penetrans* attachment (Davies & Opperman, 2006), so the interaction effect for attachment that we observe in this study may reflect variation in compatibility between collagen‐like proteins of the *P. penetrans* sources and receptors, perhaps mucin‐like proteins, expressed on the cuticles of our host lines (Davies, 2009; Phani et al., 2018). Our results support the potential for coevolution of *P. penetrans* with *M. arenaria* and the maintenance of genetic variation via negative frequency‐dependent selection, wherein *P. penetrans* populations adapt to attach to locally common genotypes of *M. arenaria* (Liu et al., 2018).

From a management perspective, the observed interaction effect supports the hypothesis that genotype‐by‐genotype interactions explain variation in the efficacy of biological control. This variation could be minimized through the use of either *P. penetrans* genotypes selected to maximize performance on local *Meloidogyne* populations or genetically diverse *P. penetrans* populations. Our second experiment evaluated this latter option by testing if attachment increased when we increased the genetic diversity of the *P. penetrans* population. We found that a combined mixture of eight *P. penetrans* sources had more than twice the rate of attachment of the mean of single parasite sources (Figure 4). Because attachment is the first step of the infection process (Sayre & Wergin, 1977), this result means that genetically diverse *P. penetrans* populations could have much higher infection rates and thus increased control of *Meloidogyne* populations, relative to *P. penetrans* populations with limited or no diversity (Channer & Gowen, 1992). In three of four host lines, the genetically diverse parasite population had higher attachment rate than the single parasite sources *on average*, but did not significantly exceed the attachment rate of at least one of the highest‐performing single sources (Table S3a, Figure S3). Thus, genetically diverse *P. penetrans* populations have reduced variation in attachment rate across host lines, relative to single parasite sources. This result supports the idea that combining multiple distinct *P. penetrans* sources increases the number of parasite genotypes with distinct host specificity, thereby increasing the probability that the parasite population contains at least one parasite genotype that can attach to the tested *M. arenaria* genotype.

In constructing a genetically diverse parasite population, we decreased the representation of any one parasite genotype relative to the low‐diversity sources. Thus, we might expect a decrease in attachment load with increased diversity, because a host may encounter a lower dose of endospores of the specific parasite genotype to which they are susceptible. Our results support this prediction: a host line's highest attachment load was consistently achieved in combination with a low‐diversity parasite source, not with the high‐diversity source (Table S3b, Figure S4). However, mean attachment load did not differ between low‐ and high‐diversity sources, pointing again to genetic diversification as a tool for reducing variation in infection outcomes. Our findings confirm those of Channer and Gowen (1992): they found that *M. incognita* and *M. javanica* hosts were less likely to acquire both very low and very high numbers of endospores when tested with mixtures of four *P. penetrans* isolates, relative to two single isolates. We expand their work in demonstrating that mixing local *P. penetrans* sources consistently and substantially increases the probability of endospore attachment on host lines established from a single field. In line with Channer and Gowen (1992)'s reasoning, we feel that this advantage of parasite diversity outweighs the cost of a reduction in maximum attachment load, because a small number of attached endospores suffices to ensure established infection (Stirling, 1984). A valuable next step would be to experimentally estimate the minimal level of parasite diversity needed to maximize attachment rate while still maintaining a sufficient load to ensure infection.

The results of our second experiment demonstrate an advantage of diverse *P. penetrans* populations against single genotypes of *M. arenaria*. Based on our first experiment, however, a field population of *M. arenaria* likely contains multiple genotypes that vary in the *P. penetrans* sources to which they are susceptible (Figures 1 and 3). We predict diverse *P. penetrans* populations to have an even greater advantage when applied to these genetically diverse field populations of *M. arenaria* (van Baalen & Beekman, 2006), similar to findings of Ganz and Ebert (2010) for a microsporidian parasite of *D. magna*. Moreover, augmenting genetic diversity of biological control populations enables rapid evolution following introduction, which facilitates establishment and ongoing adaptation to local, evolving pest populations (Hufbauer & Roderick, 2005; Szűcs et al., 2017, 2019). In conclusion, our findings establish the significance of genotype‐by‐genotype interactions in mediating the outcome of biological control and call for the use of genetically diverse populations of natural enemies to reduce variation in the efficacy of control.

## CONFLICT OF INTEREST

The authors declare no conflict of interest.

## Supporting information


Appendix S1

## Data Availability

Data for this study are available at: https://doi.org/10.5061/dryad.gmsbcc2s1.
