# Supplementary material for: A diverse parasite pool can improve effectiveness of biological control constrained by genotype‐by‐genotype interactions
Source: Evol Appl. 2022 Nov 5;15(12):2078–88. doi: 10.1111/eva.13501 (PMC9753821; doi:10.1111/eva.13501)
Supplement: Supplementary file 1 — Appendix S1 [file EVA-15-2078-s001.pdf]

SUPPLEMENTAL MATERIAL

1. Supplemental Results

1.1. Supplemental Tables of Results

1.2. Supplemental Figures of Results

2. Supplemental Methods

2.1. Establishing isofemale host lines

2.2. Rearing conditions for host lines

2.3. Field collection of parasite sources

2.4 Isolation of hosts for use in assays

2.5 Isolation of parasites for use in assays

2.6 Bioassay protocol

2.7 References

1. SUPPLEMENTAL RESULTS

1.1 Supplemental Tables of Results

Table S1. Summary of analyses for Experiment 1. Analysis of Deviance Tables (Type II tests) for fixed effects in models of (a) attachment rate, and (b) attachment load.

a. *Attachment rate, full model: (Number of hosts without endospores, number of hosts with) ~ Host line * Parasite source*

Fixed effect	*df*	χ^2	*p*
Host line	12	2264.5	<0.001
Parasite source	3	224.8	<0.001
Host line * Parasite source	33	1415.1	<0.001

b. *Attachment load, full model: Number of spores ~ Host line * Parasite source + (1|Unique ID)*

Fixed effect	*df*	χ^2	*p*
Host line	9	1425.9	<0.001
Parasite source	3	223.8	<0.001
Host line * Parasite source	18	1803.9	<0.001

Table S2. Summary of analyses for Experiment 2. Parasite diversity as a predictor of variation in (a) attachment rate and (b) attachment load.

a. *Attachment rate model: (Number of hosts without endospores, number of hosts with) ~ Diversity level + (1|Host line) + (1|Parasite source) + (1|replicate flask)*

Predictor	Level	Coefficient ± SE	z value	p value
Diversity	High	1.6 ± 0.3	4.7	<0.001
	vs. intercept-only model, ΔAIC = 8.6			

b. *Attachment load model: Number of spores ~ Diversity level + (1|Host line) + (1|Parasite source) + (1|replicate flask)*

Predictor	Level	Coefficient ± SE	z value	p value
Diversity	High	0.2 ± 0.2	0.9	0.376
	vs. intercept-only model, ΔAIC = 1.3			

Table S3. Mean attachment rate and load for low vs. high diversity parasite sources. For each of the four host lines tested, we compared the high diversity parasite source (MIX) to the eight low diversity parasite sources (P01 – P08). (a) Attachment rate is the percentage of hosts with endospores attached. (b) Attachment load is the number of endospores attached per host with one or more endospores attached. Bolding denotes the maximum attachment rate and load for each host line. Asterisks indicate a significant difference in attachment rate or load between a low diversity source and the high diversity source tested on the same host line, based on Tukey's post-hoc multiple pairwise comparison with * for p < 0.05; ** for p < 0.01; *** for p < 0.001. In (a), post-hoc comparisons were not conducted for combinations with no attachment (0%). In (b), *na* indicates combinations in which load could not be calculated because no endospores attached to hosts.

a. *Attachment rate* (mean % ± standard error)

			**Host line**			
	Diversity		H04	H08	H10	H13
**Parasite source**	*Low*	P01	20.47 ± 4.61**	30.00 ± 4.30	31.67 ± 10.76	42.50 ± 2.10***
		P02	0.00 ± 0.00	33.33 ± 3.33	26.67 ± 3.60**	46.16 ± 0.62**
		P03	0.00 ± 0.00	10.13 ± 4.40***	30.00 ± 2.72*	25.85 ± 2.51***
		P04	34.08 ± 4.81	16.67 ± 2.36***	0.00 ± 0.00	19.58 ± 7.08**
		P05	0.00 ± 0.00	32.50 ± 8.33	33.93 ± 5.02	49.52 ± 2.87*
		P06	0.00 ± 0.00	20.21 ± 6.78	24.17 ± 2.01**	39.97 ± 6.64**
		P07	0.00 ± 0.00	35.00 ± 4.19	31.67 ± 5.18	23.21 ± 1.03***
		P08	0.00 ± 0.00	30.83 ± 4.17	20.00 ± 6.94***	17.04 ± 7.19***
	High	MIX	**54.97 ± 8.99**	**45.00 ± 3.19**	**50.00 ± 4.30**	**77.50 ± 5.68**

b. *Attachment load* (mean number ± standard error)

			**Host line**			
	Diversity		H04	H08	H10	H13
**Parasite source**	*Low*	P01	1.94 ± 0.25	1.44 ± 0.08	2.35 ± 0.17	2.33 ± 0.16
		P02	*na*	1.80 ± 0.13	1.41 ± 0.10*	1.60 ± 0.14**
		P03	*na*	1.20 ± 0.20	**3.19 ± 0.25**	1.25 ± 0.09***
		P04	**3.07 ± 0.38**	1.20 ± 0.09	*na*	1.33 ± 0.33
		P05	*na*	1.49 ± 0.12	1.70 ± 0.12	**7.14 ± 0.54*****
		P06	*na*	1.25 ± 0.16	1.45 ± 0.15*	2.06 ± 0.23
		P07	*na*	**4.52 ± 0.46*****	1.68 ± 0.12	1.08 ± 0.08**
		P08	*na*	1.24 ± 0.08	1.50 ± 0.13	1.00 ± 0.00
	High	MIX	1.88 ± 0.19	1.91 ± 0.15	2.52 ± 0.15	3.06 ± 0.24

Table S4. Variation in attachment rate and load according to host genotype, parasite source, and their interaction for Experiment 2. Model comparisons for (a) attachment rate, the percentage of hosts with endospores attached, and (b) attachment load, the number of endospores attached per host with one or more endospores attached. This analysis excludes the high diversity treatment, so "Parasite source" indicates one of the eight single sources. The best model based on AIC and BIC is denoted in bold, and Weight refers to Akaike weight, a measure of relative likelihood of the model.

Model	df	AIC	ΔAIC	Weight	BIC
a. Attachment rate					
**Host line * Parasite source**	**32**	**471.7**	**0**	**1.00**	**563.0**
Host line + Parasite source	11	611.9	140.2	0.00	643.3
Host line	4	652.9	181.2	0.00	664.3
Parasite source	8	729.8	258.0	0.00	752.6
b. Attachment load					
**Host line * Parasite source**	**26**	**2176.2**	**0**	**1.00**	**2294.5**
Host line + Parasite source	12	2273.0	96.8	0.00	2327.6
Host line	5	2278.8	102.6	0.00	2301.5
Parasite source	9	2272.2	96.0	0.00	2313.1

1.2 Supplemental Figures of Results

Figure S1: Attachment rate varies with the combination of host and parasite. This figure shows data from Figure 3a in more detail. The bars represent mean attachment rates (percentage of hosts with endospores attached) across six replicates per host line * parasite source combination, and error bars show the standard error. Crosses indicate combinations for which we had no data.

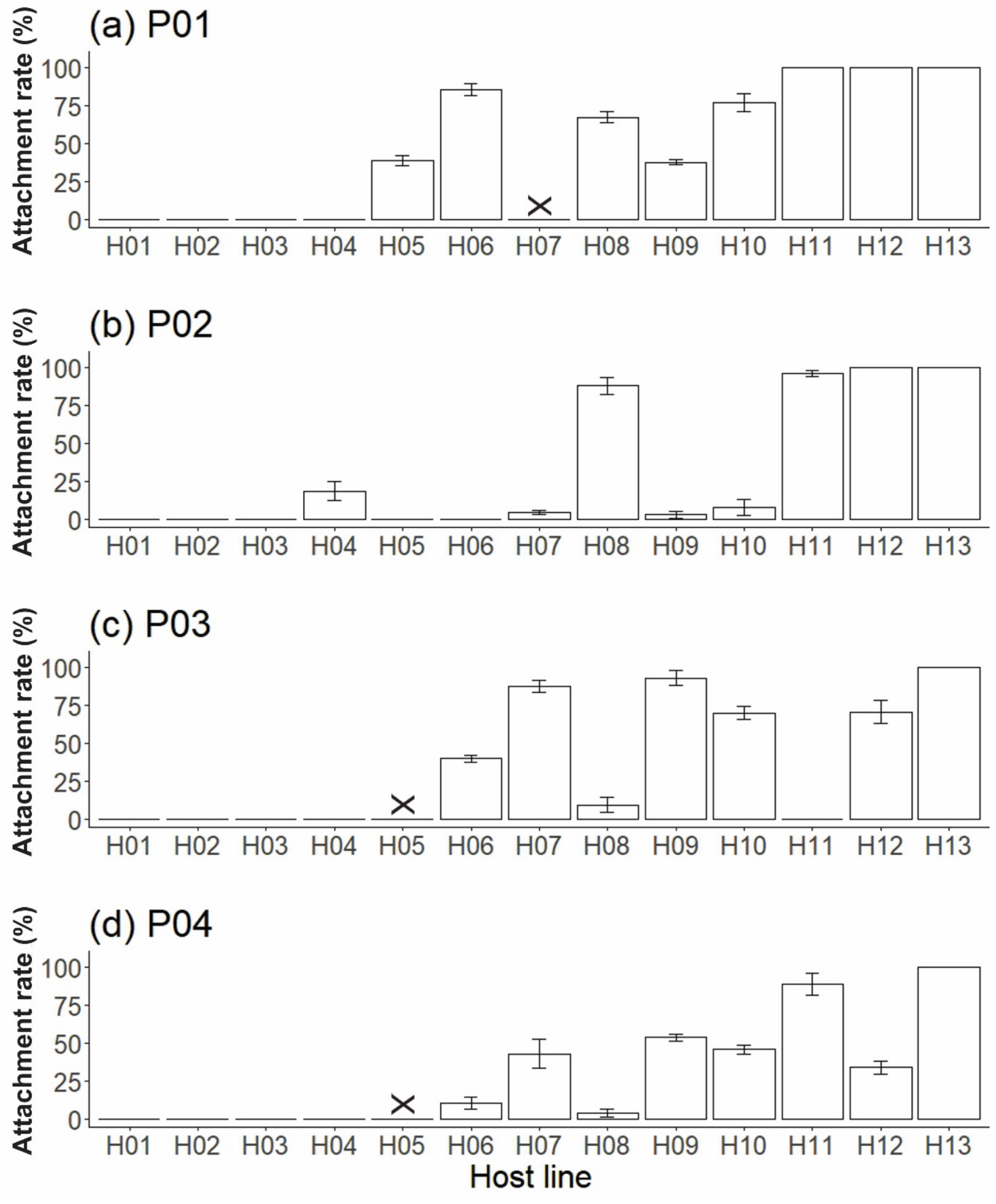

Figure S2. Attachment load varies with the combination of host and parasite. This figure shows data from Figure 3b in more detail. Boxes indicate attachment load (the number of endospores attached per host with one or more endospores attached) for each host line * parasite source combination. The boxes represent the interquartile range, the horizontal black lines indicate the medians, and whiskers extend to 25% and 75% quartiles. Black points show the number of endospores attached for individual hosts, and the blue diamond is the mean. Crosses indicate combinations for which we had no data.

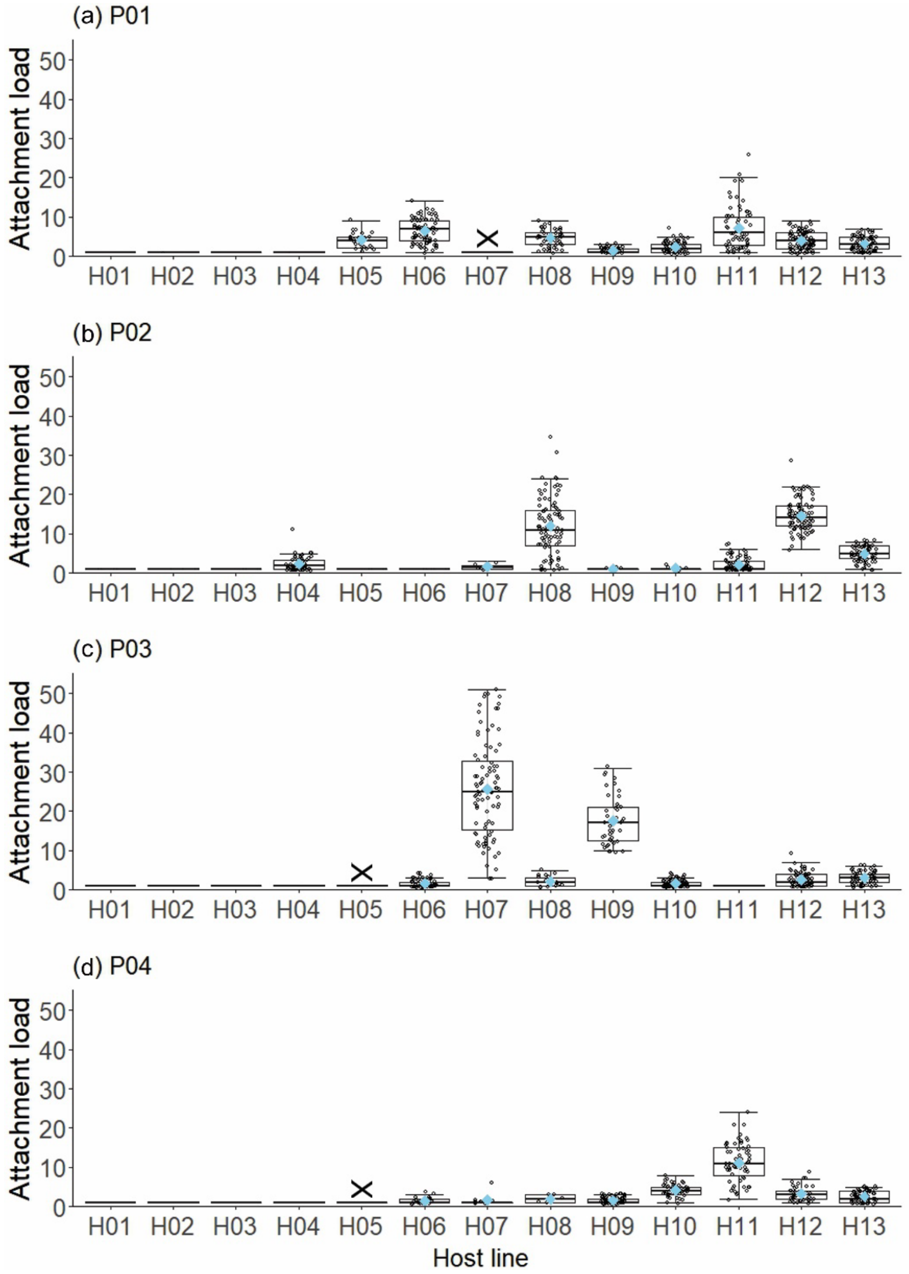

Figure S3: Increasing parasite diversity increases attachment rate. This figure shows data from Figure 4 in more detail. The bars represent mean attachment rate (percentage of hosts with endospores attached) across four replicates per host line * parasite source combination, and error bars show the standard error. MIX = the high parasite diversity source, a combination of the eight individual sources. P01 – P08 = low diversity parasite sources.

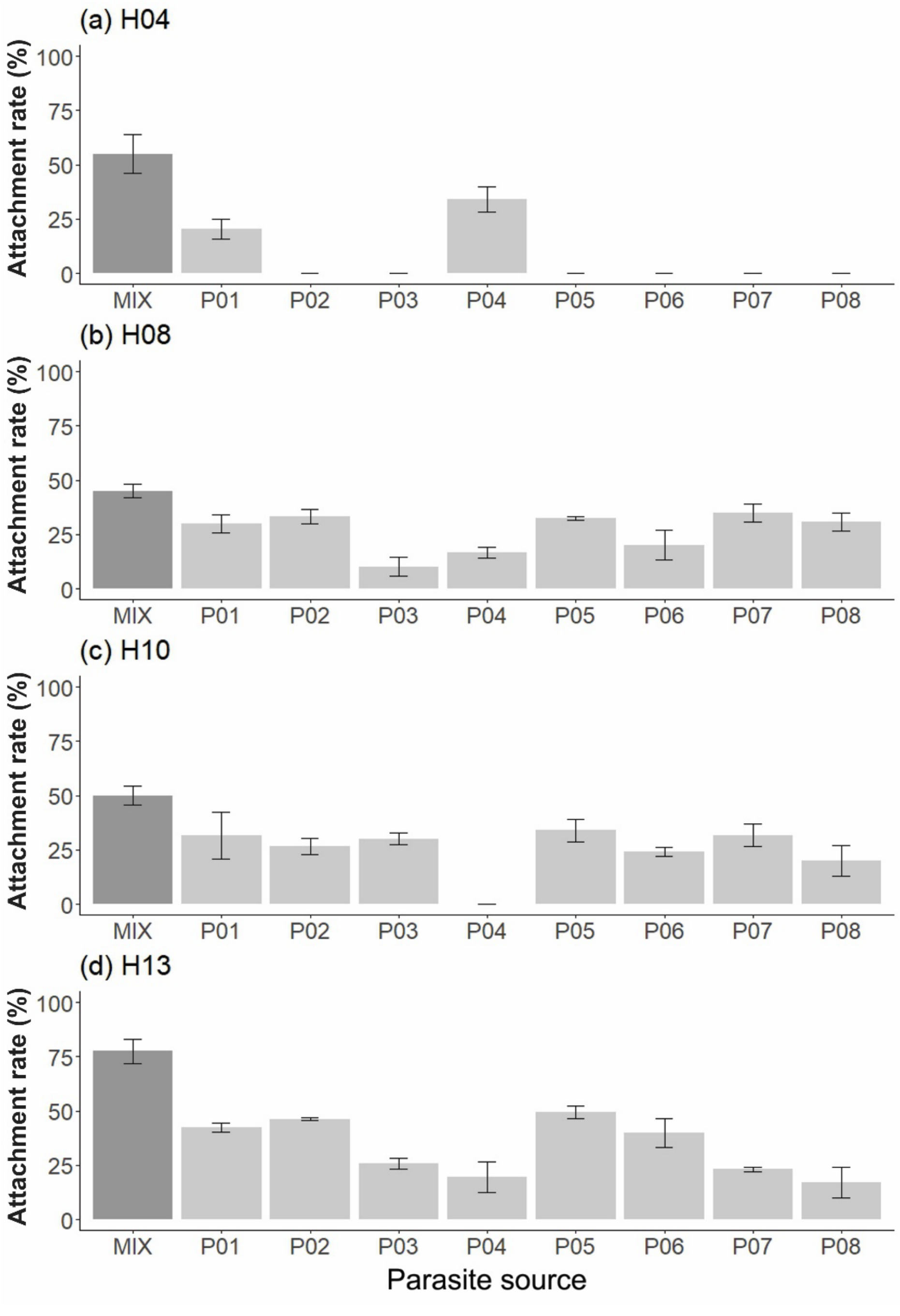

Figure S4: Increasing parasite diversity has no effect on mean attachment load. Boxes indicate attachment load (the number of endospores attached per host with one or more endospores attached) for each host line * parasite source combination. The boxes represent the interquartile range, the horizontal black lines indicate the medians, and whiskers extend to 25% and 75% quartiles. The dots show the number of endospores attached for individual hosts, and the blue diamond is the mean. MIX = the high diversity parasite source, a combination of the eight low diversity sources. P01 – P08 = low diversity parasite sources.

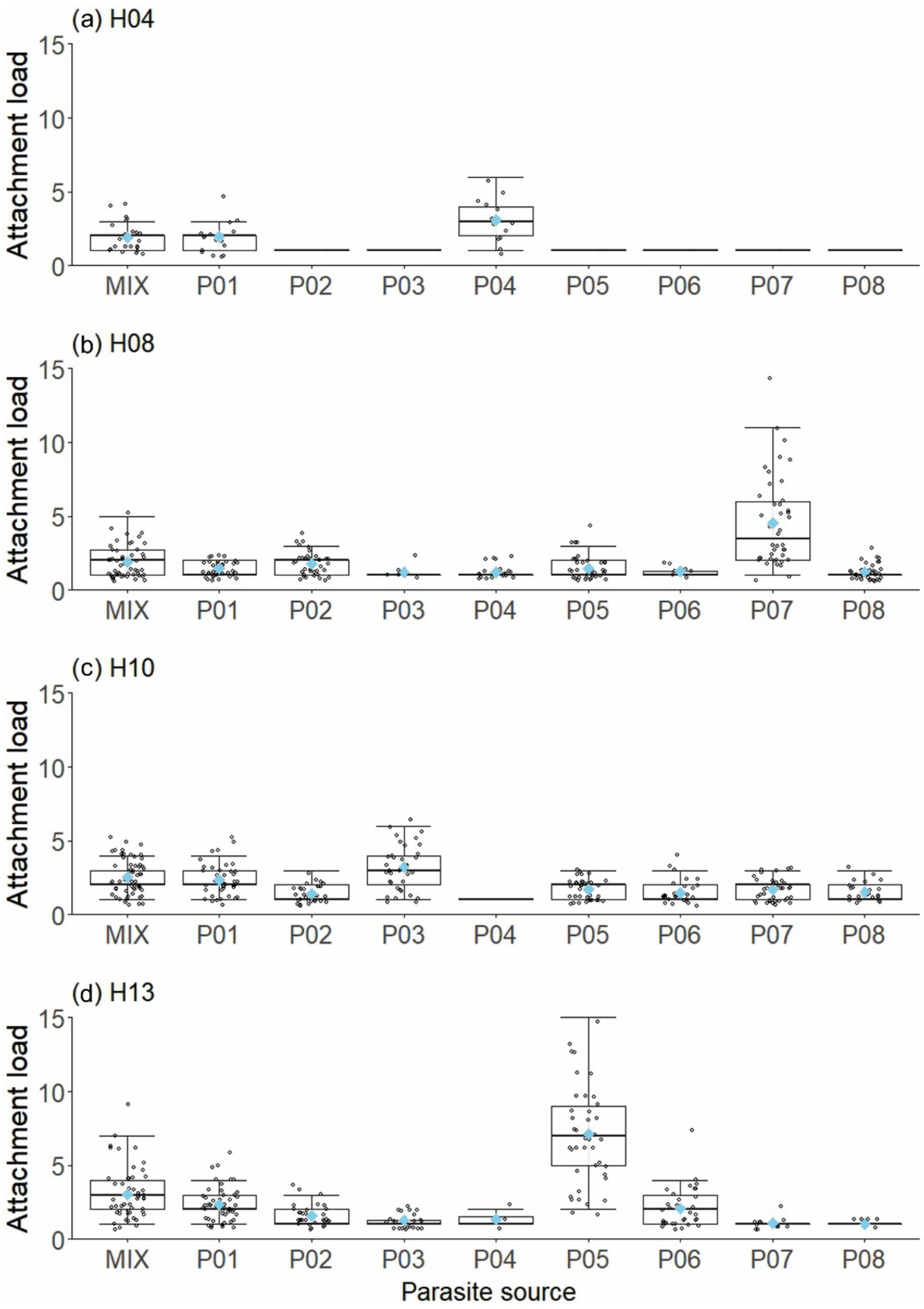

2. SUPPLEMENTAL METHODS

2.1 Establishing isofemale host lines

Isofemale lines of *Meloidogyne arenaria* were isolated from peanut roots. Whole intact root systems of peanut plants infected with *M. arenaria* were removed from the soil, rinsed in tap water, and blotted dry. The dry root system was placed in a bowl (~1000 mL capacity) containing a 20% solution of red food coloring (McCormick®) (100 mL red food coloring + 400 mL of DI water) for 15 minutes. The roots were then rinsed in tap water, blotted dry, cut into 3 – 4 cm pieces, and put in a large Petri dish with enough water to keep them moist (Thies et al., 2002). Under a stereoscope, the roots were observed and scanned for egg masses (dark maroon in Fig. S5). We used fine forceps to remove an egg mass from a gall. We avoided galls and eggs masses that were close together in order to ensure each egg mass came from a single female. Each egg mass was placed in a microcentrifuge tube with water.

To establish an isofemale line, one egg mass was inoculated into one one-month-old eggplant by depositing the egg mass in a hole in the soil at the base of the plant. We allowed isofemale lines to proliferate for approximately three months.

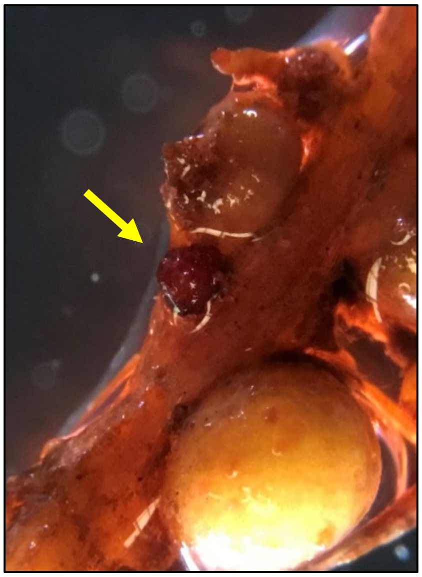

Figure S5. **Egg mass from a peanut root after staining with 20% red food coloring.** Red egg mass indicated by yellow arrow. Photo by Anne Janisch.

2.2 Rearing conditions for host lines

We used eggplants (*Solanum melongena*) to rear the isofemale lines of *M. arenaria*. The eggplants were germinated from commercialized seeds (David's Garden Seeds Eggplant Black Beauty 2477 (Black) 50 Non-GMO, Heirloom Seeds). After germination, the plants were transplanted to pots with a sterilized mix of 1:3 soil to sand and maintained in the greenhouse at a mean of 29 ºC during the day and 21 ºC at night. The plants were watered daily and received 16 hours of light. Once a week, each plant received 10 mg of 20:20:10 NPK fertilizer diluted in water. After one-month-old plants were inoculated with *M. arenaria* egg masses, the plants were maintained under the same conditions for three months. To stimulate root growth, flowers were removed.

2.3 Field collection of parasite sources

Table S5. Location of *Pasteuria penetrans* field collections

**Location name**	***P. penetrans*** **source**	**GPS coordinates**
Blackshank-Brenneman Farm	(Fig. S6a)	31.502034, -83.546682
Lower tier #1	P01	
Lower tier #3	P05	
Bowen Holbrook Farm	(Fig. S6b)	31.481739, -83.439619
Plot #1	P02	
Gibbs Farm	(Fig. S6c,d)	
Cross-Roads #1	P03	31.438192, -83.583972
Tubbs #2	P04	31.433372, -83.581230
Lang-Brenneman Farm	(Fig. S6e)	31.519693, -83.548067
Plot #A	P06	
Plot #B	P07	
Plot #D	P08	

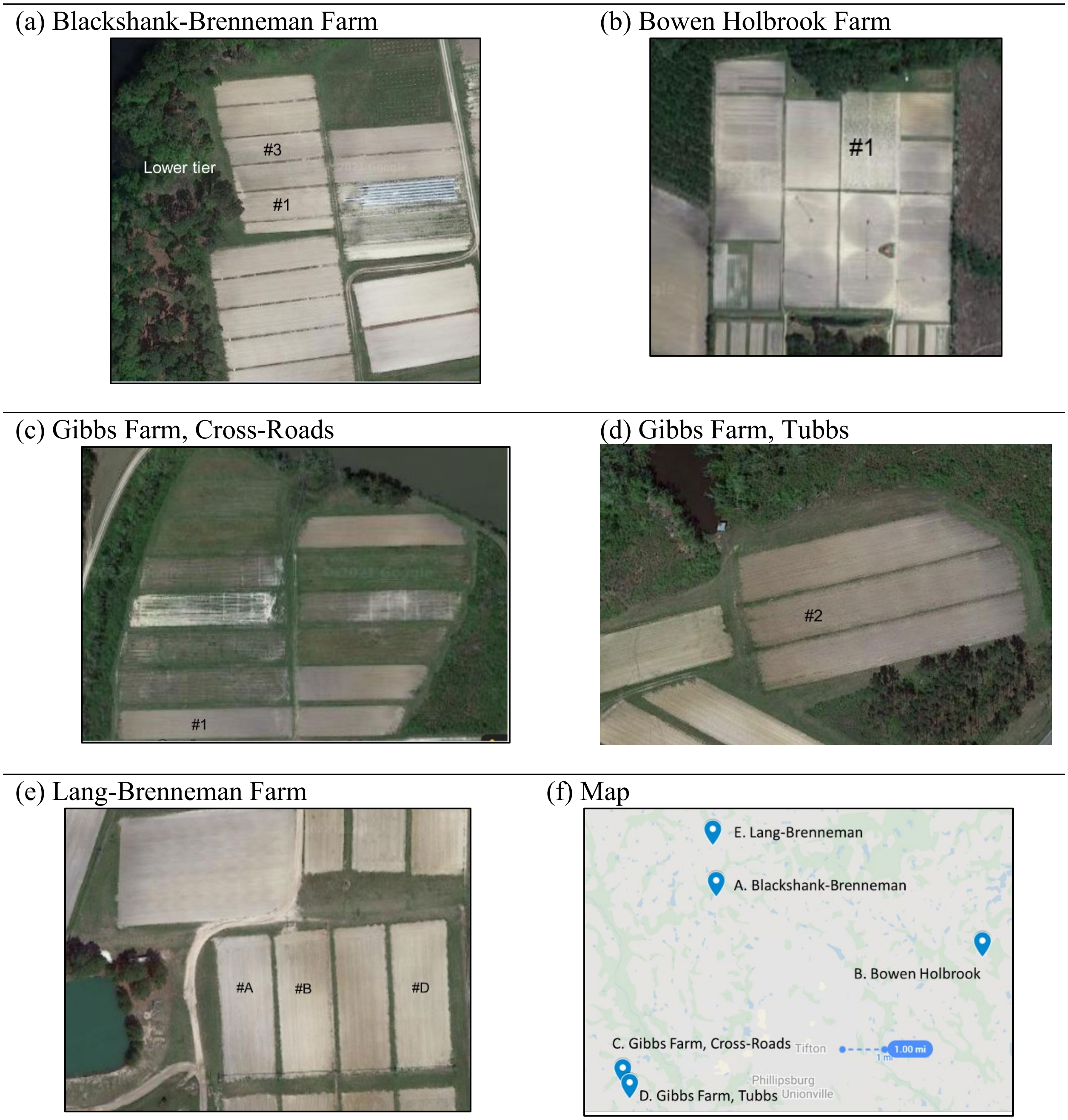

Figure S6. Location of *Pasteuria penetrans* field collections. We sampled soil with *P. penetrans* endospores from eight field sites: (a) Blackshank-Brenneman Farm, lower tier plots 1 and 3, (b) Bowen Holbrook Farm, plot 1, (c) Gibbs Farm, Cross-Roads field, plot 1, (d) Gibbs Farm, Tubbs field, plot 2, and (e) Lang-Brenneman Farm, plots A, B, and D. (f) locates each farm geographically. *Meloidogyne arenaria* isofemale lines were isolated from the Tubbs field at the Gibbs Farm.

2.4. Isolation of hosts for use in assays

We extracted nematodes eggs from eggplant roots using the Oostenbrink dish method (Oostenbrink, 1960), detailed below:

1) Chop plant roots into small pieces. Put in a blender, cover with water and blend for up to 1 minute, or until galls are broken down.
2) Place plant material on the cotton-wool milk filter placed within a sieve (Fig. S7).
3) Submerge sieve with sample gently in the water of the dish
4) Upon hatching from eggs, J2 larvae will leave the plant tissue, pass through the cotton-wool milk filter and sink to the bottom of the dish.
5) Collect J2s from the dish after 24 – 72 h and transfer to a glass beaker.
6) Let the J2s settle in the glass beaker and discard the supernatant. Pass the suspension over a 25-µm aperture sieve to reduce the volume of water.
7) Suspensions of J2s can be kept in the fridge for up to a week. Prior to use, bring to room temperature for a few hours.

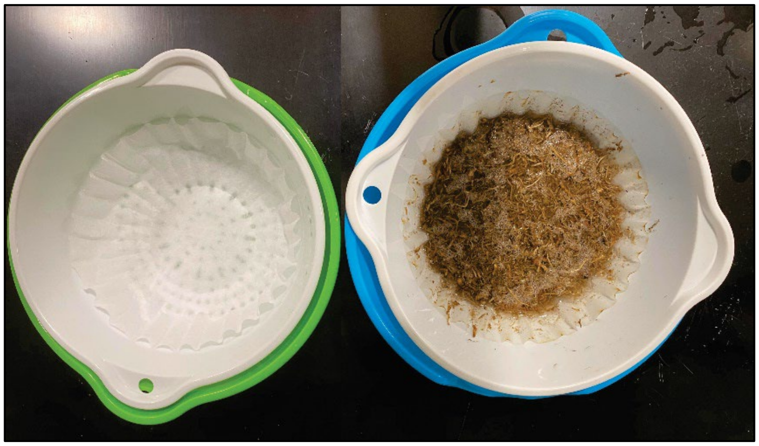

Figure S7. Oostenbrink dish. Set-up consisting of plastic dish, plastic basket and cotton-wool milk filter for extracting. Photo by Fabiane Mundim.

2.5 Isolation of parasites for use in assays

Soil containing *P. penetrans* endospores was collected from the sources identified in Supplement 2.3. We collected soil by taking eight cores (15 – 20 cm depth) from a 1x1.8-m square area in a clockwise manner from the field entry point. The soil was dried for three days at room temperature, then stored at 4 °C until the start of the experiment.

To create an inoculum of endospores from each source (adapted from Timper 2009), we:

1) Added 300 cm^3 of the dried soil sample to a 1000-ml flask.
2) Added tap water to the flask to saturate the soil, then brought the final volume to 1000 ml.
3) Sealed the flask and shook it vigorously by hand for 10 seconds.
4) Allowed soil particles settle for 5 seconds before decanting the soil–water suspension (~800 ml) into another container.
5) The above procedures were repeated until the total volume of the endospore suspension was ~15,000 mL (per parasite source), enough to be distributed across the replicate flasks.
6) The endospore suspension was shaken to homogenize, and 190 mL was added to each replicate flask (n = 6 for each host line; size: 250 cm^3).

2.6 Bioassay protocol

To evaluate attachment rate and load, we used a bioassay adapted from Timper et al., 2001.

1) 10 mL of a suspension of J2s (Supplement 2.4) were added to the flasks, followed by 190 mL of a suspension of *P. penetrans* (Supplement 2.5) to get a final volume of 200 mL.
2) Replicate flasks with J2s and endospores were placed on a rotary shaker at 180 rpm. (Fig. S8).
3) After 48 hours, J2s were extracted from the soil–water suspension by centrifugal flotation (method modified from Coolen 1979).
4) We counted attached endospores at 10 – 40x magnification with an inverted microscope.

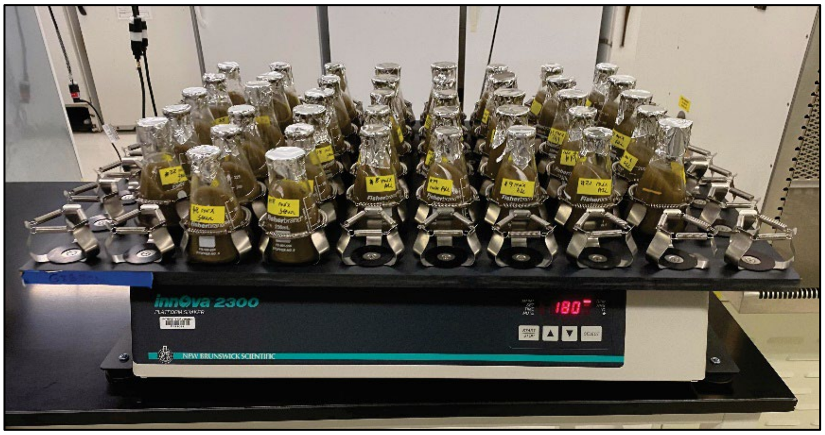

Figure S8. Bioassay setup. Flasks were shaken on a rotary shaker for 48 hours. Photo by Fabiane Mundim.

2.7 References

Coolen, W. A. (1979). Methods for the extraction of *Meloidogyne* spp. and other nematodes form roots and soil. In F. Lamberti & C. E. Taylor (Eds.), *Root-Knot Nematodes (*Meloidogyne *species) Systematics, Biology and Control* (pp. 317–329). Academic Press.

Oostenbrink, M. (1960). Estimating nematode populations by some selected methods. In J. N. Sasser & W. R. Jenkins (Eds.), *Nematology* (pp. 85–102). The University of North Carolina Press.

Thies, J. A., Merrill, S. B., & Corley, E. L. (2002). Red food coloring stain: new, safer procedures for staining nematodes in roots and egg masses on root surfaces. *Journal of Nematology*, *34*, 179–181. https://pubmed.ncbi.nlm.nih.gov/19265929/

Timper, P. (2009). Population dynamics of *Meloidogyne arenaria* and *Pasteuria penetrans* in a long-term crop rotation study. *Journal of Nematology*, *41*, 291–299. https://pubmed.ncbi.nlm.nih.gov/22736828/

Timper, P., Minton, N. A., Johnson, A. W., Brenneman, T. B., Culbreath, A. K., Burton, G. W., Baker, S. H., & Gascho, G. J. (2001). Influence of cropping systems on stem rot (*Sclerotium rolfsii*), *Meloidogyne arenaria*, and the nematode antagonist *Pasteuria penetrans* in peanut. *Plant Disease*, *85*, 767–772. https://doi.org/10.1094/PDIS.2001.85.7.767
